# Gender-specific Pathways of Peer Influence on Adolescent Suicidal Behaviors

**DOI:** 10.1177/2378023117729952

**Published:** 2017-09-07

**Authors:** Jason M. Fletcher

**Affiliations:** 1 University of Wisconsin–Madison, Madison, WI, USA

**Keywords:** peer effects, suicidal behaviors, gender, peer influence

## Abstract

The author explores new directions of understanding the pathways of peer influence on adolescent suicidal behavior by leveraging quasi-experimental variation in exposure to peer suicidal behaviors and tracing the flows of influence throughout school environments and networks. The author uses variation in peers’ family members’ suicide attempts to deploy an across–grade level, within-school analysis to estimate causal effects. Key findings include a gender-specific pathway, whereby girls are affected by their female grademates’ experiences with family member suicidality but are unaffected by their male grademates. These specific pathways allow novel approaches to be used that leverage the gender specificity of the influences within an instrumental variable analysis. The findings suggest large (gender-specific) peer effects on suicidal behaviors in adolescence.

Suicide is an important problem in the United States, its rate having steadily increased for the past 15 years (Curtin, Warner, and Hedegaard 2016), and it remains the second leading cause of death for adolescents aged 15 to 19 years (Heron 2015). Although there are a host of determinants at the individual level, such as genetic factors and the experience of psychological pain, many sociological theories strongly implicate factors outside the person, including friends, family, neighborhoods, and other contextual factors, as key determinants (Bearman 1991; Bearman and Moody 2004; Durkheim, Spaulding, and Simpson 1951; Tarde and Clark 1969).

Estimating the peer and contextual factors that determine the suicidal behaviors of adolescents is challenging. Indeed, a key set of empirical findings showing the presence of imitation as an important social process in producing suicide “clusters” has been challenged; Hoffman and Bearman (2015) show that although celebrity suicides are followed by increases in population suicidal levels (which could suggest imitation processes), the same empirical patterns are found following other newsworthy events. Furthermore, interpreting the mechanisms behind the statistical associations between the suicidal behaviors of close ties (i.e., friends) is inconclusive; indeed, Shalizi and Thomas (2011) showed that these two distinct processes, homophily and influence/contagion,^1^ are *generically confounded* in typically used observational studies.

In this article I take a step back from the specific focus on examining the dyadic effects of friends and family members on suicidal behaviors of adolescents to estimate the impacts of larger contextual structures: the effects of grademates’ exposure to family members’ suicide attempts on adolescent suicidal outcomes.

An empirical advantage of this alternative focus is the ability to deploy quasi-experimental research designs to estimate causal effects. Although using random assignment (or quasi-random assignment) strategies to examine how friends shape adolescent suicidal behaviors is unlikely to be implementable, there is a large and growing literature that uses the particular structure of U.S. junior high schools and high schools to set up a quasi-experimental research design to estimate contextual effects on behavioral outcomes (Bifulco, Fletcher, and Ross 2011; Fletcher 2010; Hoxby 2000). The key idea is that conditional on choosing a specific high school, whether a given student is assigned to 9th grade or 10th grade (and thus exposed to the peer characteristics of 9th or 10th graders in that school) is determined by the age of the student, not by other family and individual factors that may also be related the risk for suicidal behaviors of the student (i.e., confounders).

Using this across-grade, within-school design, I find a novel and important role for grademates’ experiences with family members’ suicidal behaviors in shaping adolescent behaviors. Furthermore, I show that these pathways travel within but not across gender boundaries. The heterogeneity in the results by gender suggests that female adolescents are more responsive, but not at much increased risk for exposure, to the distress of their grademates than are male adolescents and adds to the set of explanations for elevated suicide risks of female versus male adolescents. Extending these findings using a second empirical approach (an instrumental variable strategy) also allows me to provide novel evidence of the causal effects of grademates’ suicidal outcomes on own outcomes: social contagion. The estimate suggests that a 10 percent increase in the proportion of peers with suicidal thoughts increases own suicidal thoughts by more than 25 percent.

## Background Literature

This research builds on recent advances in the literature that estimate the impacts of contextual factors on suicidal behaviors. Much research has been devoted to examining the effects of friends and family on these behaviors in adolescents, but separating how much of the mechanism linking these behaviors is determined by influence, contagion, and mimicry or by homophily and selection remains a challenge. Some of the more persuasive evidence has used longitudinal network data to consider whether the timing of the patterns in the data is consistent with influence. For example, Abrutyn and Mueller (2014) selected a sample of adolescents who did not report suicidal behaviors at baseline (wave 1 in the National Longitudinal Study of Adolescent to Adult Health [Add Health]) and asked whether having a friend who attempted suicide between data collection waves was associated with the emergence of suicidal thoughts in the ego at follow-up (wave 2). Although the authors included a variety of statistical controls to attempt to limit the likelihood that homophily explains these associations, the empirical design could not conclusively rule out this mechanism (Cohen-Cole and Fletcher 2008; Shalizi and Thomas 2011).

Indeed, the findings of Abrutyn and Mueller (2014) are likely the most compelling evidence on the topic of friend and contextual influences on suicidal behaviors of adolescents. Most earlier research either used cross-sectional data without a credible causal research design or used longitudinal data but did not fully consider the issue of timing and reverse causality as strongly as did Abrutyn and Mueller (e.g., Liu 2006; Winfree and Jiang 2010).

Although much of the previous literature focus on impacts of close friends, family, or celebrities, the current study expands the measurement of contextual factors by focusing on “meso-level” associations in suicidal behaviors: I estimate the impacts of suicide attempts of grademates’ family members. There is much evidence that classmate or grademate characteristics can influence adolescent outcomes (Bifulco et al. 2011; Fletcher 2010), which suggests that these “meso” exposures may not be too distal to be important behavioral predictors.

In addition to examining the main effects of exposure to grademates’ family members’ suicidal behaviors, I also examine potential heterogeneity of the effects. There are very large sex differences in suicidal behaviors (Eaton et al. 2012; Eisenberg and Resnick 2006), though there are several possible explanations. Female teenagers may be especially susceptible to peer influence because they have closer friendship ties than male teenagers (Crosnoe 2000), so that the effects of contagion or mimicry could be especially large for women. Alternatively, other scholars (e.g., Kral 1994) present evidence that would suggest higher levels of suicide for women because they have higher rates of psychological pain (which is consistent with sex differences in depression). Girls and women may also be at higher risk through an increased likelihood of exposure to peer suicidal behaviors, because their close friends are more likely to be female.

This research also contributes to the literature assessing the causal effects of peer behaviors on own behaviors, so-called endogenous peer effects (Manski 1993). I follow some of the literature in addressing key challenges in separating these behavioral spillovers from other group-level processes or simultaneity bias by using school fixed effects combined with an instrumental variable strategy. Fletcher (2010) used a similar strategy to show peer influence in smoking behaviors between adolescents and showed that including school fixed effects reduced the estimates substantially (see also Kim and Fletcher [forthcoming], who show evidence of spillovers in adolescent criminal activities). Leveraging the contextual effect of grademates’ exposure to family-level suicidal behaviors and assuming that the only pathway of influence between this exposure and own suicidal behaviors is through grademates’ suicidal behavior change allows a causal estimate of peer influence in suicidal behaviors.

## Data

I use data from the restricted version of Add Health. Add Health is a school-based, longitudinal study of the health-related behaviors of adolescents and their outcomes in young adulthood. Beginning with an in-school question-naire administered to a nationally representative sample of students in grades 7 through 12 in 1994 and 1995, the study followed up with a series of in-home interviews of students approximately 1 year, 6 years, and 13 years later. By design, the Add Health survey included a sample stratified by region, urbanicity, school type, ethnic mix, and size. Add Health is especially attractive because it contains a variety of socioemotional and noncognitive outcomes.

In Add Health, 20,745 students were surveyed during wave 1. I drop approximately 1,000 observations because of missing data on grade level at wave 1 (~600 observations), suicidal outcomes (~200 observations), or exposure to family or friend suicide (~100 observations). The key exposure measure is to grademates’ family members’ suicide attempts, measured using the yes/no question “Have any of your family tried to kill themselves during the past 12 months?” The primary outcome measure is suicidal thoughts, measured using the yes/no question “During the past 12 months, did you ever seriously think about committing suicide?”

Table 1 presents selected summary statistics. Thirteen percent of the students report suicidal thoughts in the previous year. Four percent of the sample report having family members who attempted suicide in the previous year, but the proportion at the grade level is highly variable, ranging from 0 percent to 100 percent, with a standard deviation of 4 percent.

As discussed above, the research design deployed in the analysis uses school-level fixed effects and thereby focuses on across-grade and within-school variation in exposure to grademates’ family members’ suicide attempts. The key assumption for the research design is that, conditional on school, the variation in grademate exposure is quasi-randomly assigned. Table A1 reports results consistent with this assumption. The “balancing test” is in the spirit of tests for successful randomization in the randomized control trial literature, in which researchers compare baseline characteristics between individuals in the “treatment” and “control” groups; a lack of association between treatment status and these characteristics is viewed as evidence for successful randomization. Similarly, I estimate associations between predetermined student-level variables and their level of exposure to grademates’ family members’ suicide attempts. Column 1 shows evidence that quasi-randomization is not a valid assumption in general in these data, in that regression analysis without fixed-effects estimates associations between several student characteristics and their “treatment” status (i.e., grademate levels of family members’ suicide attempts). Column 2 then shows that these associations are eliminated by the inclusion of school fixed effects; these results are consistent with the assumption used to leverage a quasi-experimental research design.

## Results

Table 2 presents baseline ordinary least squares and school-level fixed-effects regression analysis linking grademate characteristics (i.e., exposure to family members’ suicide attempts) on own measures of suicidal thoughts in wave 1 of the survey. The results show clear evidence of same-sex peer influences and no evidence of opposite-sex peer influences. Table 3 shows that these effects are particularly strong for girls. A large set of sociodemographic variables are controlled but not shown. The results suggest that a 10 percent increase in exposure to female *grademates* with these family experiences is related to an increase in own suicidal thoughts of 2.3 percentage points (from a base rate of 13 percent). Importantly, these impacts are net of school-level fixed effects.^2^

As a next step, I use an instrumental variable analysis to estimate causal effects of peer suicidal behaviors on own suicidal behaviors. This examination differs from Tables 2 and 3 because it shifts attention from estimating the effects of peer characteristics (i.e., peer exposure to their own family’s suicidal behaviors^3^) to estimating the effects of peer behaviors (i.e., contagion). The key additional assumption to leverage an instrumental variable specification is that grademates’ family exposure effects own suicidal behaviors through the channel of peer suicidal behaviors (i.e., the exclusion restriction). Another way of thinking about the difference between Table 2 and Table 4 results is that the former is in the spirit of an intent-to-treat analysis, and the latter is in the spirit of a treatment-on-the-treated analysis.

Table 4 begins in column 1 with a “naive” ordinary least squares regression linking peer suicidal thoughts with own suicidal thought outcomes. As is well known in the peer effect literature, this specification suffers from many empirical problems, including unobserved group-level factors (confounders) and simultaneity bias (the reflection problem) (Manski 1993). Column 2 solves the reflection problem by using an instrumental variable strategy, whereby grademates’ exposure to family suicide attempts is assumed to affect own suicidal thoughts through its effect on grademates’ suicidal thoughts. The estimate suggests that a 10 percent increase in the proportion of peers with suicidal thoughts increases own suicidal thoughts by 3.6 percentage points (from a base of 13 percent). The first-stage *F* statistic in column 3 is greater than 24, suggesting a strong instrument. As a point of reference, the estimated association between reporting a friend who attempted suicide and own suicidal thoughts is 16 percentage points. Column 4 repeats the instrumental variable analysis with the addition of school-level fixed effects to control for unobserved confounders at the school level. Surprisingly, the results suggest limited scope for possible school-level confounders, as the peer effect coefficient is reduced by less than 0.1 percentage point for a 10 percent change in peer behaviors. Column 5 shows that the first-stage *F* statistic is greater than 10, which indicates a strong instrument.

A potential limitation with the instrumental variable results in Table 4 stems from the gender-specific effects of grademates’ exposure presented in Table 3. That is, the instrument in Table 4 combines both same- and opposite-sex grademates’ exposure in a single measure, but Table 3 suggests that only same-sex grademates’ exposure is associated with own suicidal thoughts. Table 5 pursues this issue further by using same-sex grademates’ exposure as the instrumental variable of interest. Using same-sex grademates rather than all grademates as the instrument also has the advantage of allowing additional sets of fixed effects to be used to further reduce confounding. Column 1 repeats results from Table 4, column 4 (i.e., inclusion of school fixed effects) but uses same-sex grademates as the instrument. The results suggest larger peer spillovers in suicidal thoughts with this instrument: a 10 percent increase in peer suicidal thoughts increases the likelihood of own suicidal thoughts by nearly 5 percentage points. Column 3 adds School × Grade Level fixed effects, which should serve to further reduce confounding factors; School × Grade fixed effects are available in this specification because each grade is now “divided” into a female and male grade with respect to the instrument, so the comparisons in each grade are between male and female adolescents.^4^ The results become even larger, with a 10 percent increase in peer suicidal thoughts predicted to increase own thoughts by nearly 7 percentage points. Finally, column 5 pursues a different set of fixed effects, using School × Sex effects rather than School × Grade effects. The idea here is divide each school into a “male” school and “female” school and then compare each sex within their own “schools.” These fixed effects control for confounding specific to each gender group at each school. The results suggest that a 10 percent increase in exposure to same-sex grademates’ suicidal thoughts increases own suicidal thoughts by approximately 5 percentage points.

## Conclusion

This research makes several contributions to the literature examining social influences on adolescent suicidal behaviors. Estimating peer influence faces considerable challenges due to confounding, endogeneity of peers, and simultaneity bias (i.e., peer influence is reciprocal). Using a quasi-experimental approach, I estimate causal effects of exposure to peers’ (grademates’) family members’ suicide attempts on own suicidal thoughts. The findings show relatively large effects, and the pathways are gender specific, where female grademates’ experiences affect girls but not boys, and male grademates’ experiences affect boys more than girls. These results contribute a new explanation for the elevated rates of suicidal behaviors in adolescent girls than in boys.

An additional assumption (i.e., an exclusion restriction) allows the use of an instrumental variables specification that pushes further to elicit the causal effects of peer suicidal behaviors on own outcomes. The results suggest large spillover effects that imply the possibility that policies that affect the suicidal behaviors of one student could positively spill over to reduce suicidal behaviors of (untreated) classmates.

An important question for future research include understanding why girls seem to react more strongly to, and why boys are largely inoculated from, exposure to meso-contextual effects in the form of grademates who experience family member’s suicides and why this contextual effect appears to operate only through gender-specific pathways.

## Figures and Tables

**Table 1:** Selected Descriptive Statistics, Add Health, Wave 1 (*n* ≈ 19,700).

Variable	*M*	*SD*	Minimum	Maximum
Suicidal thoughts, W1	0.13	0.34	0	1
Grade-level suicidal thoughts, W1	0.13	0.07	0	1
Grade-level family suicide attempt	0.04	0.04	0	1
Same-sex grade-level family suicide attempts	0.04	0.05	0	1
Opposite-sex grade-level family suicide attempts, W1	0.05	0.05	0	1
Black	0.22	0.42	0	1
Hispanic	0.17	0.37	0	1
Other race	0.08	0.27	0	1
Age (years), W1	16.13	1.72	12	21
Male	0.50	0.50	0	1
Maternal education, W1	13.18	2.25	0	17
Family income (×$10,000), W1	4.55	3.96	0	99
Parents married, W1	0.70	0.42	0	1
Family suicide attempt indicator	0.04	0.21	0	1
Friend suicide attempt indicator	0.17	0.38	0	1
Missing family information indicator	0.34	0.47	0	1
Grade 8 at W1	0.13	0.34	0	1
Grade 9	0.18	0.38	0	1
Grade 10	0.20	0.40	0	1
Grade 11	0.19	0.39	0	1
Grade 12	0.17	0.37	0	1

*Note:* W1 = wave 1.

**Table 2:** Regression Analysis Linking Grademates’ Exposure to Family Suicide Attempts and Own Suicidal Thoughts: Baseline Ordinary Least Squares and School-level Fixed-Effects Analysis.

Variable	Suicidal Thoughts at W1		
	No Fixed Effects	School Fixed Effects	School Fixed Effects
Grade-level family suicide attempts, W1	.120** (.057)	.093 (.068)	
Same-sex grade-level family suicide attempts, W1			.180*** (.054)
Opposite-sex grade-level family suicide attempts, W1			−.017 (.051)
Family member attempted suicide, W1	.144*** (.014)	.143*** (.014)	.145*** (.014)
Friend attempted suicide, W1	.164*** (.008)	.162*** (.008)	.161*** (.009)
Black	−.029*** (.006)	−.030*** (.008)	−.032*** (.008)
Hispanic	−.011 (.007)	−.013 (.008)	−.014 (.008)
Other race	.018** (.009)	.005 (.011)	.006 (.011)
Age, W1	.006* (.003)	.008** (.003)	.007** (.003)
Male	−.045*** (.005)	−.046*** (.005)	−.041*** (.006)
Maternal education, W1	−.000 (.001)	−.001 (.001)	−.001 (.001)
Family income, W1	−.000 (.001)	−.000 (.001)	−.000 (.001)
Married parents, W1	−.017*** (.006)	−.016*** (.006)	−.016*** (.006)
Parent is happy, W1	−.032** (.015)	−.034** (.015)	−.032** (.015)
Constant	.060 (.050)	.042 (.051)	.048 (.051)
Observations	19,725	19,725	19,413
*R*^2^	.061	.068	.069
*F*	4.458	1.837	11.20

*Note:* Additional controls: grade-level fixed effects, constant, indicator for missing family-level variables. Standard errors clustered at the school level. W1 = wave 1.

**Table 3:** Regression Analysis Linking Grademates’ Exposure to Family Suicide Attempts and Own Suicidal Thoughts: Baseline Ordinary Least Squares and School-level Fixed-effects Analysis by Gender.

Variable	Suicidal Thoughts W1	
	School Fixed Effects	School Fixed Effects
Sample	Male	Female
Same-sex grade-level family suicide attempts, W1	.083 (.074)	.230*** (.083)
Opposite-sex grade level family suicide attempts, W1	.040 (.067)	−.050 (.100)
Family member attempted suicide, W1	.163*** (.025)	.134*** (.017)
Friend attempted suicide, W1	.143*** (.015)	.168*** (.012)
Black	−.040*** (.009)	−.026** (.012)
Hispanic	−.018* (.010)	−.009 (.015)
Other race	.010 (.014)	.000 (.018)
Age, W1	.010** (.004)	.005 (.005)
Maternal education, W1	−.002 (.001)	−.001 (.002)
Family income, W1	−.000 (.001)	−.000 (.001)
Married parents, W1	−.005 (.008)	−.028*** (.009)
Parent is happy, W1	−.016 (.019)	−.045** (.020)
Constant	−.055 (.061)	.085 (.082)
Observations	9,547	9,866
*R*^2^	.064	.077
*F*	1.258	7.629

*Note:* Additional controls: Grade level fixed effects, indicator for missing family level variables. Standard errors clustered at the school level.

**Table 4:** Instrumental Variable Analysis.

Outcome	Suicidal Thoughts				
Sample	Full	Full	Full	Full	Full
Specification	Basic	IV	First stage	IV/FE	First stage
Grade-level suicidal thoughts, W1	.096** (.047)	.361*** (.118)		.352* (.182)	
Black	−.027*** (.006)	−.024*** (.006)	−.015*** (.004)	−.030*** (.008)	−.001 (.001)
Hispanic	−.010 (.007)	−.009 (.006)	−.006 (.005)	−.012 (.008)	−.003* (.001)
Other race	.017** (.009)	.015* (.009)	.007 (.006)	.006 (.011)	−.002 (.002)
Age, W1	.006* (.003)	.006* (.003)	−.001 (.001)	.007** (.003)	.002* (.001)
Male	−.045*** (.005)	−.045*** (.005)	.001 (.001)	−.046*** (.005)	.000 (.001)
Maternal education, W1	−.000 (.001)	−.001 (.001)	.000 (.000)	−.001 (.001)	−.000 (.000)
Family income, W1	−.000 (.001)	−.000 (.001)	.000 (.000)	−.000 (.001)	.000 (.000)
Married parents, W1	−.018*** (.006)	−.017*** (.006)	−.003* (.001)	−.016*** (.006)	−.001 (.001)
Parent is happy, W1	−.031** (.015)	−.031** (.014)	−.001 (.003)	−.033** (.015)	−.002 (.003)
Family member attempted suicide, W1	.144*** (.014)	.142*** (.014)	.003 (.002)	.142*** (.014)	.003 (.002)
Friend attempted suicide, W1	.163*** (.008)	.161*** (.008)	.007*** (.002)	.162*** (.008)	.002 (.001)
Missing family information indicator	−.002 (.006)	−.001 (.006)	−.003* (.001)	−.001 (.006)	−.001 (.001)
Grade-level family suicide attempts, W1			.331*** (.067)		.264*** (.082)
Constant	.054 (.050)	.026 (.051)	.096*** (.018)		.064*** (.014)
Observations	19,726	19,724	19,724	19,724	19,724
*R*^2^	.062	.059	.113	.052	.086
*F*			24.65		10.45

*Note:* Grade fixed effects controlled but omitted from the table. Standard errors are clustered at the school level. FE = fixed effects; IV = instrumental variable; W1 = wave 1.

**Table 5:** Instrumental Variable Analysis: Use of Same-sex Grademate Instrument.

Variable	Suicidal Thoughts					
	Full	Full	Full	Full	Full	Full
	IV/School FE	First Stage	IV/School × Grade FE	First Stage	IV/School × Sex FE	First Stage
Same-sex grade-level suicidal thoughts, W1	.493*** (.100)		.690*** (.114)		.518*** (.102)	
Black	−.031*** (.008)	.000 (.002)	−.031*** (.008)	.001 (.001)	−.031*** (.008)	.000 (.001)
Hispanic	−.013 (.008)	−.000 (.002)	−.013 (.009)	.002 (.002)	−.014 (.008)	.000 (.002)
Other race	.005 (.011)	−.000 (.002)	.005 (.011)	.001 (.002)	.005 (.011)	−.001 (.002)
Age, W1	.008** (.003)	.000 (.001)	.008** (.003)	−.002** (.001)	.008** (.003)	.000 (.001)
Male	−.015** (.007)	−.056*** (.006)	−.003 (.007)	−.055*** (.006)		
Maternal education, W1	−.001 (.001)	−.000 (.000)	−.001 (.001)	−.000 (.000)	−.001 (.001)	−.000 (.000)
Family income, W1	−.001 (.001)	.000 (.000)	−.001 (.001)	.000 (.000)	−.001 (.001)	.000 (.000)
Married parents, W1	−.017*** (.006)	.000 (.002)	−.017*** (.006)	.002 (.001)	−.017*** (.006)	−.000 (.001)
Parent is happy, W1	−.032** (.015)	−.001 (.003)	−.033** (.015)	−.000 (.002)	−.033** (.015)	−.001 (.003)
Family member attempted suicide, W1	.139*** (.014)	.007** (.003)	.140*** (.014)	.011*** (.004)	.139*** (.014)	.008** (.003)
Friend attempted suicide, W1	.158*** (.009)	.007*** (.002)	.159*** (.009)	.002 (.002)	.159*** (.009)	.004** (.002)
Missing family information indicator	−.002 (.006)	.001 (.002)	−.002 (.006)	.002 (.001)	−.003 (.006)	.000 (.001)
Same-sex grade-level family suicide attempts, W1		.283*** (.052)		.321*** (.063)		.286*** (.056)
Constant		.105*** (.016)		.168*** (.012)		.075*** (.014)
Observations	19,655	19,655	19,655	19,655	19,655	19,655
*R*^2^	.045	.177	.010	.215	.029	.068
*F*		29.61		26.27		25.86
Number of schools, W1	144	144				

*Note:* Grade fixed effects controlled but omitted from the table. Standard errors are clustered at the school level. FE = fixed effects; IV = instrumental variable; W1 = wave 1.

## Footnotes

1 Alternatively, these processes are called social selection versus social influence.

2 Table 2A in the Appendix examines nonlinear effects of grademates’ exposure to family suicidal behaviors. For girls, the results suggest impacts concentrated at relatively high levels of exposure but no effects otherwise. The pattern for boys is less clear.

3 This classification implicitly assumes that the grademate’s behaviors did not cause the family member’s suicide attempt.

4 This comparison is reflected in the first-stage results in column 6, where the “male” coefficient shows that male adolescents in general are exposed to same-sex exposures that are 5.5 percentage points lower than female adolescents’ exposures. The assumption necessary for these fixed effects is that there are no opposite-sex grademate spillovers on own behaviors. This difference in exposure is also worthy of future study. Mueller and Abrutyn (2014) showed that suicidal behavior disclosure between friends is a key element underlying social contagion in these outcomes. Assuming that families with male adolescents have similar likelihoods of experiencing suicide as families with female adolescents, the difference in reports of family exposure suggest that male adolescents do not seek out information about family members’ suicide attempts to the same degree that female adolescents do and thus are at lower risk for passing along this information to others.

## Appendix

**Table A1:** Balancing Tests Are Consistent with Quasi-random Variation in Grademate Exposure.

Fixed Effects?	Grade-level Family Suicide Attempt	
	None	School
Black	.002 (.003)	−.000 (.001)
Hispanic	.000 (.002)	−.000 (.001)
Other race	−.000 (.003)	−.000 (.001)
Age, W1	.001 (.001)	.000 (.000)
Male	−.000 (.001)	−.000 (.000)
Maternal education, W1	−.001** (.000)	−.000 (.000)
Family income, W1	−.000*** (.000)	−.000 (.000)
Married parents, W1	−.003*** (.001)	−.001 (.001)
Parent is happy, W1	−.001 (.002)	−.001 (.002)
Family member attempted suicide, W1	.003 (.002)	−.002 (.003)
Friend attempted suicide, W1	.001 (.001)	.000 (.001)
Missing family information indicator	.000 (.001)	.000 (.001)
School-level family suicide attempt		.486 (.336)
Constant	.041*** (.011)	.016 (.017)
Observations	19,752	19,752
*R*^2^	.027	.277

*Note:* Grade fixed effects controlled but omitted from the table. Standard errors are clustered at the school level. W1 = wave 1.

**Table A2:** Regression Analysis Linking Grademates’ Exposure to Family Suicide Attempts and Own Suicidal Thoughts: Nonlinear Analysis.

Outcome	Suicidal Thoughts		
Sample	Full	Male	Female
Fixed Effects?	School	School	School
Second quartile grade-level family suicide attempts (>0 and <0.04)	.008 (.008)	.014 (.010)	.002 (.012)
Third quartile grade-level family suicide attempts (>0.04 and <0.0625)	.005 (.009)	.010 (.013)	.002 (.013)
Fourth quartile grade-level family suicide attempts (>0.0625)	.012 (.009)	.007 (.011)	.020 (.013)
Black	−.030*** (.008)	−.039*** (.009)	−.025* (.013)
Hispanic	−.013 (.008)	−.020* (.010)	−.007 (.015)
Other race	.005 (.011)	.008 (.013)	.000 (.018)
Age, W1	.008** (.003)	.011*** (.004)	.006 (.005)
Male	−.046*** (.005)		
Maternal education, W1	−.001 (.001)	−.002 (.001)	−.001 (.002)
Family income, W1	−.000 (.001)	−.000 (.001)	−.000 (.001)
Married parents, W1	−.016*** (.006)	−.005 (.008)	−.028*** (.009)
Parent is happy, W1	−.034** (.015)	−.021 (.018)	−.045** (.020)
Family member attempted suicide, W1	.143*** (.014)	.161*** (.025)	.131*** (.017)
Friend attempted suicide, W1	.162*** (.008)	.145*** (.014)	.169*** (.012)
Missing family information indicator	−.002 (.006)	.000 (.007)	−.005 (.008)
Observations	19,725	9,772	9,953
*R*^2^	.069	.064	.077
*F*	.745	.614	.883

*Note:* Additional controls: grade-level fixed effects, constant. Standard errors clustered at the school level. W1 = wave 1.
